# PrEP Use Willingness and Perceptions Among Young Female Sex Workers in Kisumu, Kenya: Implications for HIV Prevention

**DOI:** 10.1007/s10461-026-05122-5

**Published:** 2026-04-11

**Authors:** Dan Omollo, Pam Murnane, Judith Ayallo, Jacob Onyango, Sheri D. Weiser

**Affiliations:** 1 Impact Research and Development Organization, Kisumu, Kenya; 2 Department of Epidemiology & Biostatistics, University of California San Francisco, San Francisco, CA USA; 3 Sustainable Development for HIV Health (SD4H), University of California San Francisco, San Francisco, CA USA; 4 Department of Medicine, University of California San Francisco, San Francisco, CA USA

**Keywords:** Female sex workers, Pre-exposure prophylaxis, Willingness, Kenya

## Abstract

Female sex workers (FSW) are disproportionately burdened with HIV. Pre-exposure prophylaxis (PrEP) is highly effective in preventing HIV transmission, yet determinants of willingness to use PrEP among young FSW is less understood. We assessed factors associated with willingness to use PrEP, using data from a cross-sectional study on PrEP adherence strategies, among HIV-negative FSW aged 18–24 years living in Kisumu City, Kenya. We characterized differences in demographics and sexual history by awareness of PrEP using Pearson’s chi-square test. Among FSW aware of PrEP, we further assessed factors associated with willingness to use PrEP. We enrolled 200 FSW, of whom 83% reported being aware of PrEP. Among those aware of PrEP, 16% had previously taken PrEP. On average, for each additional day or night spent away from the neighborhood in the past month, FSW had 12% decreased odds (AOR = 0.88, 95%CI: 0.77, 0.99) of willingness to use PrEP. While most FSW in Kisumu City were aware of PrEP and perceived it positively, uptake was hindered by stigma, discomfort with health services, and lifestyle factors like mobility. To close the gap between awareness and actual use of PrEP, there is need for flexible and mobile PrEP delivery models tailored to the unique lifestyles of FSW, such as community-based outreach, peer delivery, or digital health interventions that support care continuity despite mobility.

## Introduction

Globally, female sex workers (FSW) carry a disproportionate burden of HIV, with estimates suggesting that FSW are 14 times more likely to be living with HIV compared to women in the general population [1]. Among FSW, structural and contextual factors such as poverty, gender inequality, violence, stigma, and criminalization of sex work contribute to limited agency in preventing HIV acquisition [2, 3].

Pre-exposure prophylaxis (PrEP) is a biomedical intervention that provides additional HIV prevention options beyond traditional methods for at risk populations [4–8]. Increasing FSW’s access to routine pre-exposure prophylaxis (PrEP) is part of global commitments to provide PrEP to those at highest risk [9–11]. PrEP is highly effective against HIV infection when taken as prescribed; however, questions about FSW’s willingness to use PrEP still remain. Rates of uptake, adherence and retention have proved lower than hoped in many PrEP trials and demonstration projects among FSW and other high-risk populations in sub-Saharan Africa [12–14]. New PrEP users often struggle integrating daily PrEP use into their routine practice [15, 16], are negatively affected by family and peers’ opinions [17], and fear the pill will be confused for ART [18] hence people will think they are HIV positive. Conversely, some women find PrEP acceptable and integrate it into their normal schedule with ease [19].

In September 2015, the World Health Organization (WHO) recommended PrEP for all persons at high risk of HIV acquisition [20]. Although PrEP is gaining recognition as a critical component of comprehensive HIV programming, the scale up of its uptake among high-risk populations such as FSW is generally slow in SSA [21]. In July 2016, the Ministry of Health (MOH) in Kenya released guidelines which recommended initiation of PrEP for HIV-negative persons with substantial ongoing risk of HIV infection [22]. National PrEP roll out in Kenya officially launched in May 2017 and has since expanded in a variety of care centres like Drop-in-Centres (DiCEs; i.e. safe spaces where FSW and other key populations access HIV prevention, care and treatment services) [14]. Although more than 900 public and private health facilities have been equipped to offer PrEP to FSW in Kenya, little is understood regarding the experiences and perceptions towards PrEP use among FSW in this population [23].

Understanding the experiences and perceptions of FSW regarding PrEP use is critical for informing interventions to support PrEP uptake and adherence in this high risk and vulnerable population [24]. We assessed awareness, willingness and perceptions of PrEP use among FSW in Kisumu County, western Kenya using data from a cross-sectional study conducted in 2022.

## Methods

### Design Setting and Purpose

This was a cross-sectional study conducted to inform the design and implementation of combination prevention interventions, including PrEP for FSW, in Kisumu, Kenya. The study was conducted through Impact Research and Development Organization in Kisumu. We explored the feasibility and acceptability of delivering PrEP as part of a defined package of HIV prevention interventions to FSW and to assess structural, interpersonal, individual and method-related facilitators and barriers to initiation and sustained use of PrEP.

### Participants

We aimed to recruit a target sample of 200 young FSW between July and October 2022. This sample size was determined based on feasibility (expected number of eligible participants accessible within the study sites and timeframe) and precision considerations. Assuming a proportion of 50% for key outcomes (to yield the maximum variance), a sample of 200 provides margin of error of ± 7% at the 95% confidence level for prevalence estimates. To be eligible, participants had to: (1) self-identify as females or transgender women born males who have sex with men; (2) self-report being HIV-negative or unknown status; (3) be between 18 and 24 years of age; (4) be a resident of or working in the study community; (5) report having exchanged sex for goods, money, favors or other services in past 3 months; and (6) be able to complete study procedures in English, Kiswahili or Dholuo languages. We excluded FSW who were unable to verify their age, were cognitively impaired or were intoxicated. FSW were purposively sampled and recruited from pre-specified locations (*n* = 13) identified as “hotspots” which included bars, hair salons and social clubs or other community venues where FSW often meet their male clients. Recruitment was facilitated by two trained peer educators familiar with each location, and the approach was convenience-based within these predefined hotspots. The study sample was distributed across bars (*n* = 133), brothels (*n* = 28), hair salons (*n* = 22), and social clubs (*n* = 17), reflecting the primary venues where FSW congregate and ensuring representation across different sex work typologies. It was determined a priori based on feasibility and the need to capture diverse perspectives among FSW, consistent with formative and implementation research standards where sample adequacy is guided by information power and conceptual saturation rather than statistical power.

### Data Collection

We used best practices [25] to develop data collection forms which were then subjected to rigorous pilot testing prior to data collection. Eligible FSW provided written informed consent processes with trained research assistants who assured them of their confidentiality. Those who consented were then taken through a 30–45 minutes’ structured questionnaire on socio-demographic profile, sexual behavior characteristics, and PrEP use experiences and perceptions.

### Measures

*Socio-demographic Characteristics* Respondents were asked their age, educational attainment, marital status, ethnicity, and typical income from sex with paying and non-paying clients.

*Sexual behaviour characteristics* FSW were asked to report their sexual behaviour during their last paid sex which included condom use, lubricant use, anal sex and intoxication before or during sex.

*PrEP use willingness and perceptions* Participants were asked whether they had previously heard about PrEP, if they had taken PrEP, and from what sources they learned about PrEP. We also asked how likely it is for them to take PrEP to lower their chances of getting HIV or recommend it to a HIV- negative partner (1 = extremely unlikely to 6 = extremely likely). On a 4-point Likert scale (1 = strongly disagree, 4 = strongly agree), participants also rated their comfort level in seeking PrEP from a health care provider, concerns that people would think they have HIV if taking PrEP, and comfort in disclosing to their main partner or main client if taking PrEP. Finally, we asked where participants would be comfortable receiving a PrEP prescription and the number of days or nights they had spent away from the neighbourhood where they live within the past 30 days. For regression analysis, *PrEP use willingness* was operationalized from the item asking how likely participants were to take PrEP to lower their chances of getting HIV. Responses on the 6-point Likert scale (1 = extremely unlikely to 6 = extremely likely) were dichotomized, with scores of 4–6 coded as “willing to use PrEP” and scores of 1–3 coded as “not willing to use PrEP.”

### Statistical Analysis

To describe sexual behaviour, we compared FSW aware and not aware of PrEP and summarized PrEP awareness by key socio-demographic and sexual behaviour characteristics using t-test (for continuous variable) and chi-square test (for categorical variables). Willingness to use PrEP was treated as a binary outcome variable, derived from the 6-point Likert item as described above. Among FSW aware of PrEP, we compared PrEP use willingness and perceptions between FSW who ‘ever’ and ‘never’ used PrEP before.

We performed multivariate logistic regression to determine factors associated with PrEP use willingness among young FSW in Kisumu City who are aware of PrEP. We included in our analysis demographic factors (age, education level, marital status, and income) and sexual behavior factors (condom use at last paid sex, number of clients in the past month, alcohol or substance use before sex, and engagement in anal sex). These factors were selected based on prior evidence showing their association with PrEP awareness, willingness, or use among female sex workers in sub-Saharan Africa and other settings [12, 13, 17]. We checked the linearity of log odds by examining plots of each of the predictor variables against the log odds of PrEP use willingness. Collinearity of predictors was assessed and collinear predictors were not included the regression analysis. All analyses used Stata, version 18 (Basic Edition 1985–2023 StataCorp LLC, Texas 77845 USA) and p-value level of significance was set at 0.05.

## Results

### Demographic Characteristics of Participants

We recruited 200 FSW from bars (133), brothels (28), hair salons (22) and social clubs (17) into this study. Although eligibility criteria included both cisgender and transgender women, all participants enrolled in this study self-identified as cisgender female sex work. Table 1 displays their demographic and sexual behavior data grouped by awareness of PrEP, most of which were comparable by PrEP awareness. Overall, the median age of FSW was 22 years (IQR: 20–23) and median age of sexual debut was 18 years (IQR: 16–19). Almost all FSW were born in Kenya (98.5%), and at the time of the study, 98% were primarily residing in Kisumu County and 85% were of Luo ethnic community. The majority (78.5%) had completed at least basic (primary and/or secondary) education. Only 3% were married, 8% were living together as married, and 89% were not in a union (i.e., single). Overall, 65.0% of participants reported earning less than $10 per client, compared to 35.0% who reported earning $10 or more. The only significant difference observed was in the use of lubricants during the last sexual encounter, with 57 (34.3%) of PrEP-aware individuals reporting lubricant use compared to 5 (14.7%) among those unaware of PrEP.

**Table 1:** Demographic and sexual behavior characteristics of young FSW in Kisumu by PrEP awareness

Characteristic	Aware of PrEP (*n* = 166) n (%)	Unaware of PrEP (*n* = 34) n (%)	Total (*N* = 200) n (%)	*p*-value*
Age: Median (IQR)	22 (21–23)	22 (20–23)	22 (20–23)	0.6132
Sexual debut age: Median (IQR)	18 (16–19	18 (16–19)	18(16–19)	0.9930
Education				
None, or only literacy	1 (0.6)	0 (0)	1 (0.5)	0.0872
Basic education	151 (91)	32 (94.1)	183 (91.5)	
Tertiary	14 (8.4)	2 (5.9)	16 (8)	
Marital status				
Currently married	5 (3.0)	1 (2.9)	6 (3.0)	0.2592
Living together as married	15 (9.1)	1 (2.9)	16 (8.0)	
Not in union	146 (87.9)	32 (94.2)	178 (89)	
Ethnic community				
Luo	139 (83.7)	31 (91.2)	170 (85.0)	0.2683
Luhya and others	27 (16.3)	3 (8.8)	30 (15.0)	
Country of birth				
Kenya	164 (98.8)	33 (97.1)	197 (98.5)	0.4505
Uganda and others	2 (1.2)	1 (2.9)	3 (1.5)	
Income (amount) typically received for having sex with a client ($)				
Below $ 5	59 (35.5)	17 (50.0)	76 (38.0)	0.0531
$ 5–10	44 (26.5)	10 (29.4)	54 (27.0)	
$ 10–20	33 (19.9)	6 (17.7)	39 (19.5)	
Above $ 20	30 (18.1)	1 (2.90)	31 (15.5)	
Type of sex (last sex) with paying client				
Vaginal	157 (94.6)	32 (94.1)	189 (94.5)	0.9145
Anal/ both vaginal & anal	9 (5.4)	2 (5.9)	11 (5.5)	
Used condom (last sex)	149 (89.8)	29 (85.3)	178 (89.0)	0.4484
Used lubricant (last sex)	57 (34.3)	5 (14.7)	62 (31.0)	0.0241
Intoxicated during last sex	67 (40.4)	9 (26.5)	76 (38.0)	0.1284

### Sexual Behavior Characteristics

Most (89.0%) FSW reported using a condom during their last sexual encounter with a paying client and 31% FSW used lubricants during their last sex. A little more than a third (38.0%) reported being intoxicated with alcohol or other drugs before sex at their last sexual intercourse with a client. The last sexual encounter with a paying sex partner was mostly (95%) vaginal with a majority (61.3%) of FSWs reporting sex with a partner of unknown HIV status (Table 1).

### PrEP Awareness

Of those surveyed, 166 (83.0%) reported being aware of PrEP prior to participation in the survey, of which 27 (16.3%) had previously taken PrEP. Non-governmental organizations were mentioned as the single most common (17.5%) source of information about PrEP, followed by radio and/or television (12.7%), other female sex workers (10.9%) and then healthcare providers (9.0%). However, most FSW mentioned having heard about PrEP from at least several sources, with just a few mentioning single sources.

### PrEP Use Experiences and Perceptions Among FSW Aware of PrEP

As previously stated, 166 FSW had awareness of PrEP but of those, only twenty-seven (16.3%) reported having previously taken PrEP (Table 2). Of those who reported having taken PrEP, 19 (70.4%) were on PrEP during the period of the survey. Generally, most (82.5%) FSW said they were likely to take PrEP in the future (30.7% “extremely likely”, 33.7% “very likely”, and 18.1% “somewhat likely”). Most of the FSW (83.5%) perceived PrEP as a good method of protection against HIV (63.9% “strongly agree” and 19.9% “agree”). About half (51.8%) would disclose to their main partner if they were taking PrEP but only 22.3% would do that to a male client. Most FSW (68.7%) would recommend PrEP to HIV-negative partners (“extremely likely” =21.1%, “very likely” =25.9% and “somewhat likely” =21.7%). However, the majority (74.6%) of FSW expressed discomfort in seeking PrEP from healthcare providers (“strongly disagree” =45.1%, “disagree” =29.5%) and more than a third (38.6%) had concerns that people would think they have HIV if it is known they are taking HIV medicine (“strongly agree” =17.5% and “agree” =21.1%).

Among FSW previously aware of PrEP, 63.3% preferred to access PrEP at a public health facility as compared to other facilities that provide services to FSW such as drop-in centers (21.1%) or other community locations (4.8%), but this differed significantly between FSW ever and never on PrEP (*p* = 0.001). However, there were no significant differences between FSW ever and never on PrEP in terms of their level of comfort in seeking PrEP from healthcare providers (*p* = 0.398), likelihood of disclosing PrEP use to their main partner or male client (*p* = 0.772), taking PrEP to lower their chances of getting HIV (p-0.432) and recommending PrEP to a HIV-negative partner (*p* = 0.736). The level of concern that other people would think FSW have HIV if taking PrEP and belief that taking a daily HIV medicine would be a good way to protect someone from getting HIV also did not differ across the two groups (Table 2).

**Table 2:** PrEP use willingness and perceptions among FSW aware of PrEP(N=166)

Characteristic	Ever on PrEP (*n* = 27) *n* (%)	Never on PrEP (*n* = 139) *n* (%)	Total (*n* = 166) *n* (%)	*p*-value
Likelihood of disclosing PrEP use to main partner				
Yes	15 (55.6)	71 (51.1)	86 (51.8)	*p* = 0.771
No	12 (44.4)	66 (47.5)	78 (47.0)	
Refused/don’t know	0 (0)	2 (1.4)	2 (1.2)	
Likelihood of disclosing PrEP use to male clients				
Yes	7 (25.9)	29 (20.9)	36 (21.7)	*p* = 0.772
No	20 (74.1)	109 (78.4)	129 (77.7)	
It depends	0 (0)	1 (0.7)	1 (0.6)	
Comfort seeking PrEP from healthcare provider				
Strongly disagree	12 (44.4)	63 (45.3)	75 (45.1)	*p* = 0.398
Disagree	5 (18.5)	44 (31.7)	49 (29.5)	
Agree	4 (14.8)	16 (11.5)	20 (12.1)	
Strongly agree	6 (22.2)	15 (10.8)	21 (12.7)	
Refused/ Don’t know	0 (0)	1 (0.7)	1 (0.6)	
Likelihood of taking PrEP to lower her chances of getting HIV				
Extremely unlikely	0 (0)	14 (10.1)	14 (8.5)	*p* = 0.142
Very unlikely	0 (0)	9 (6.5)	9 (5.4)	
Somewhat unlikely	0 (0)	6 (4.3)	6 (3.6)	
Somewhat likely	4 (14.8)	26 (18.7)	30 (18.1)	
Very likely	11 (40.7)	45 (32.4)	56 (33.7)	
Extremely likely	12 (44.4)	39 (28.1)	51 (30.7)	
Likelihood of recommending PrEP to HIV-negative partner				
Extremely unlikely	5 (18.5)	18 (13.0)	23 (13.9)	*p* = 0.736
Very unlikely	3 (11.1)	15 (10.8)	18 (10.9)	
Somewhat unlikely	1 (3.7)	8 (5.8)	9 (5.4)	
Somewhat likely	9 (33.3)	27 (19.4)	36 (21.7)	
Very likely	5 (18.5)	38 (27.3)	43 (25.9)	
Extremely likely	4 (14.8)	31 (22.3)	35 (21.1)	
Refused/Don’t know	0 (0)	2 (1.4)	2 (1.2)	
Would be concerned that people will think they have HIV if taking an HIV medicine				
Strongly disagree	15 (55.6)	43 (31.0)	58 (35.0)	*p* = 0.062
Disagree	7 (25.9)	37 (26.6)	44 (26.5)	
Agree	3 (11.1)	32 (23.0)	35 (21.1)	
Strongly agree	2 (7.4)	27 (19.4)	29 (17.5)	
Taking a daily HIV medicine would be a good way to protect myself from getting HIV				
Strongly disagree	4 (14.8)	8 (5.8)	12 (7.2)	*p* = 0.432
Disagree	1 (3.7)	13 (9.3)	14 (8.4)	
Agree	6 (22.2)	27 (19.4)	33 (19.9)	
Strongly agree	16 (59.3)	90 (64.8)	106 (63.9)	
Refused/Don’t know	0 (0)	1 (0.7)	1 (0.6)	
Preferred access point for PrEP				
Public Health Facilities	13 (48.2%)	22 (15.8%)	35 (21.1%)	*P* = 0.001
Drop-in-Centres (DiCE)	8 (29.6%)	97 (69.8%)	105 (63.3%)	
Community	2 (7.4%)	6 (4.3%)	8 (4.8%)	
Refused/Don’t know	4 (14.8%)	14 (10.1%)	18 (10.8%)	

### Factors Associated with Willingness to Use PrEP Among FSW

In bivariate logistic regression analysis, FSW mobility (number of days or nights spent away from the neighborhood within the past 1 month) (OR = 0.90, 5%CI: 0.84, 0.97) and history of having had sex with a HIV-infected individual (OR = 0.13, 95%CI: 0.02, 0.81) were found to be associated with willingness to use PrEP among FSW in Kisumu City. However, in multivariate analysis, with all potential factors included and adjusting for age and level of education, only mobility of the FSW was found to be associated with their willingness to use PrEP. On average, for each additional day or night spent away from the neighborhood within the past one month, FSW had 12% decreased odds (AOR = 0.88, 95%CI: 0.77, 0.99) of willingness to use PrEP. (Table 3).

**Table 3:** Factors associated with willingness to use PrEP among FSW aware of PrEP in Kisumu City (*N* = 166)

Characteristic	Crude OR (95%CI) *	*P*-value	Adjusted OR (95%CI) **	*P*-value
Age, per year	1.02 (0.80, 1.29)	0.881	0.92 (0.58, 1.44)	0.712
Education (any versus none)	1.42 (0.30, 6.64)	0.660	1.30 (0.12, 14.38)	0.828
Marital status (single versus other)	1.23 (0.34, 4.50)	0.757	1.11 (0.09, 1.56)	0.713
Condom use (last sex_)	0.60 (0.13, 2.79)	0.517	0.89 (0.87, 9.00)	0.919
Lubricant use (last sex)	0.59 (0.26, 1.33)	0.203	0.16 (0.02, 1.18)	0.072
Being intoxicated before sex (last sex)	0.80 (0.36, 1.80)	0.590	1.27 (0.27, 5,86)	0.763
Mobility (no of nights away in the past 1 month)	0.90 (0.84, 0.97)	0.005	0.88 (0.77, 0.99)	0.042
History of having sex with HIV infected client	0.13 (0.02, 0.81)	0.029	0.11 (0.01, 4.16)	0.237
History of forced sex	2.12 (0.60, 7.40)	0.309	1.18 (0.02, 6.47)	0.201

* Odds Ratio obtained from univariate logistic regression

** Odds Ratio obtained from a multivariate logistic regression model adjusting for all variables in table

## Discussion

PrEP awareness was high among this cohort of young FSW, consistent with other recent studies conducted in western Kenya [26] and Tanzania [15]. Specific socio-demographic or sexual behavior characteristics were not associated with this high PrEP related awareness among FSW. This high level of PrEP awareness in hyper-endemic settings may be indicative of effective public heath messaging to such key populations in the region [26]. Non-governmental organizations, radio and television shows as well as other FSWs and healthcare providers have collectively played a big role in creating public health awareness with regards to PrEP as a suitable HIV prevention intervention for these young FSW.

Despite the high level of awareness among FSW in Kisumu, less than a third had initiated PrEP by the time of the survey. This low PrEP initiation trend was similarly observed among study samples of FSW in western Kenya [13] and Dar-es-Salaam [15] in 2019 and 2022 respectively. The observed gap between high awareness and low uptake suggests that knowledge alone is insufficient to drive PrEP use among FSW. Stigma related to HIV medicine, misconceptions about its side effects, competing livelihood priorities, and fragmented service delivery models may collectively discourage initiation even among those well-informed about PrEP. Similarly, among FSW who had initiated PrEP, a considerable proportion (nearly one-third) was no longer using PrEP. PrEP continuation is a proxy indicator of effective PrEP use. Stigma (fear of being viewed to be HIV positive) and health system related challenges (discomfort accessing PrEP from health care providers) are some of the documented explanations for low PrEP continuation [13]. Another study in Congo in 2021 reported that the main reasons for declining PrEP among FSW included not being interested in PrEP and the feeling that PrEP is unnecessary [27].

In our study sample, engagement with high-risk sexual behavior was common. The proportion of condom use with paid clients was higher than what has been reported among other studies with sex workers [28, 29]. However, this rate was found to be much lower with their regular partners which can be attributed to a sense of trust and common generalized perception that HIV risk is low with regular partners [28, 30–33]. However, such perceptions may increase vulnerability given that many regular partners also have other concurrent partners. Addressing this misconception requires couple-centered counseling, risk communication that normalizes PrEP use within intimate relationships, and integration of partner testing or mutual disclosure initiatives. The rate of use of lubricants was also low with those aware of PrEP being twice as likely to use lubricants as those not aware of PrEP. Although there is still dearth of information regarding use and preferences of lubricants by FSW before or during sex [34], recent studies have emphasized that use of lubricants result in improved sexual health and well-being [35, 36]. Despite reporting such risky sexual behaviour as having multiple sex partners who also have other multiple partners, being intoxicated before sex and practicing anal sex, over a half of the FSW aware of PrEP were not taking PrEP at the time of the survey.

We found a significant difference in preferred place of PrEP provision between FSW ever and never on PrEP. About half of FSW ever on PrEP tended to prefer Drop-in-Centres (DiCEs) somewhat similar to what was reported by a study in Malawi [37], while the majority of those never on PrEP mentioned public health facilities as their preferred place of PrEP provision. DiCEs were preferred by FSW ever on PrEP probably due to the manner in which FSW are handled and type of information they get as they access health services from there. This suggests that support to increase FSW’s willingness to engage with healthcare providers and partners about PrEP may be useful for successful implementation of PrEP rollout in a region with high HIV prevalence.

Although most FSW expressed willingness to use PrEP, willingness did not consistently translate into actual initiation or continuation. Willingness appears influenced by individual-level perceptions of HIV risk and trust in PrEP efficacy, whereas actual use is more strongly affected by contextual and structural barriers such as mobility, stigma, and discomfort accessing services at health facilities. This distinction highlights the need for interventions that bridge the gap between motivation and behavior—such as peer navigation, mobile delivery platforms, and stigma-sensitive provider training [13]. Notably, just slightly more than half were comfortable disclosing to their main partners if they were using it. Willingness would stem from some factors such as perception of HIV risk and fear of social stigma [38]. In this study, accessing PrEP from a healthcare provider was reported as a major concern to the majority of FSW as well as the social stigma for fear of being perceived to be HIV positive. Similar findings were reported in a study conducted in western Kenya in 2019 [13].

Our findings were contrary to what other studies already found with regards to relationship between PrEP awareness and willingness [39, 40]. While both FSW mobility and a history of sexual contact with HIV-positive individuals were significantly associated with willingness to use PrEP in bivariate analysis, only mobility remained significantly associated with decreased willingness to use PrEP in the multivariate model. This finding suggests that highly mobile FSW may face structural or psychosocial barriers that limit their engagement with preventive health services like PrEP. Even though some HIV prevention information reach mobile FSW than their non-mobile counterparts [41], mobility generally disrupts access to consistent healthcare, reduce opportunities for PrEP counseling, or reflect a more transient lifestyle that deprioritizes long-term health planning [42, 43]. The observed association between having had sex with an HIV-positive individual and willingness to use PrEP in bivariate analysis may reflect heightened risk perception among this subgroup of FSW. Direct exposure to a known HIV-positive partner could increase awareness of personal vulnerability, prompting greater motivation to adopt preventive strategies such as PrEP. Similar findings have been reported in studies among key populations in sub-Saharan Africa, where perceived HIV risk strongly predicts willingness to use PrEP [12, 13, 44]. However, this association lost significance in the multivariate model, possibly due to overlapping behavioral and structural factors—such as stigma, inconsistent access to health services, or mobility—that jointly influence prevention behaviors. We did not find a significant association between PrEP use willingness and marital status, condom use, lubricant use, being intoxicated during sex and having history of forced sex. Although the odds ratios pointed towards a given pattern, the confidence intervals were too wide to allow us discern a significant relationship.

Our findings align with Kenya’s National HIV Prevention Strategy (2023–2028) and the Kenya PrEP Implementation Framework, both of which emphasize expanding PrEP access among key populations such as female sex workers. However, the gap between awareness and actual uptake, particularly among women with regular partners, highlights ongoing challenges in demand creation, risk perception, and service accessibility. Strengthening tailored counseling to address low perceived risk and implementing flexible, mobile-friendly PrEP delivery models could better accommodate FSW mobility and enhance the effectiveness of national frameworks in achieving sustained PrEP use.

Our findings should also be interpreted in light of the study’s methodological design. Participants were recruited through purposive, convenience-based sampling across hotspot venues such as bars, salons, and social clubs, with recruitment led by 2 trained peer educators familiar with these networks. The study could therefore be biased due to our reliance on recruiting from networks and physical spaces that are known to the study team to include sex workers; as there may be other networks and spaces that the study team does not access/know of. The target sample of 200 FSW was distributed across these sites based on FSW availability. While this strategy enabled efficient recruitment of a diverse group of young FSW and facilitated peer trust, it may limit generalizability to other settings where recruitment dynamics differ.

### Limitations

Since many of our measures were self-report, recall and self-report bias may have affected our findings, particularly if participants were more inclined to admit to favorable views about future PrEP use. Despite including transgender women in the eligibility criteria, none were enrolled. Future studies should use targeted approaches to recruit transgender women to better understand their unique PrEP experiences and needs. While the sample size of 200 limited statistical power for detecting smaller associations in regression analyses, it was sufficient to provide stable estimates for key descriptive and exploratory comparisons. Lastly, since the study was carried out on a young and relatively homogenous population, our results may not be generalizable to other populations with different demographic characteristics. However, we prioritized this young age group due to their extremely elevated risk of HIV acquisition.

## Conclusion

In this study among young female sex workers in Kisumu, Kenya, we found high awareness of PrEP but relatively low initiation and continuation rates. Although most participants expressed willingness to use PrEP, mobility emerged as a barrier, with more mobile FSW showing reduced willingness to initiate PrEP. Discomfort accessing PrEP from healthcare providers and stigma related to perceived HIV status also hindered uptake. These findings underscore the need for differentiated, mobile-friendly PrEP delivery models that integrate stigma reduction and provider sensitization. Tailored interventions that build on existing awareness while addressing mobility and structural barriers may enhance PrEP uptake and sustainment among young FSW in high HIV-burden settings.

## Data Availability

Data is available from the authors upon reasonable request.

## References

[1] Baral S, et al. Reconceptualizing the HIV epidemiology and prevention needs of female sex workers (FSW) in Swaziland. PLoS ONE. 2014;9(12):e115465. 10.1371/journal.pone.0115465 PMC4274078 25531771

[2] Shibesh BF, et al. Uptake of retroviral pre-exposure prophylaxis and its associated factors among female sex workers, Northwest Ethiopia. AIDS Res Therapy. 2023;20(1):77. 10.1186/s12981-023-00573-5 PMC10626692 37926821

[3] Leyna G, Mmbaga EJ. *Predictors of mHealth use in promoting adherence to pre-exposure prophylaxis among female sex workers: an evaluation of the Jichunge intervention in Dar es Salaam, Tanzania.* 2022. 10.1186/s12913-022-08245-2 PMC9254514 35787285

[4] Walters SM, et al. Awareness of pre-exposure prophylaxis (PrEP) among women who inject drugs in NYC: the importance of networks and syringe exchange programs for HIV prevention. Harm Reduct J. 2017;14(1):40. 10.1186/s12954-017-0166-x PMC5492910 28662716

[5] Baeten JM, et al. Antiretroviral prophylaxis for HIV prevention in heterosexual men and women. N Engl J Med. 2012;367(5):399–410. 10.1056/NEJMoa1108524 PMC3770474 22784037

[6] Choopanya K, et al. Antiretroviral prophylaxis for HIV infection in injecting drug users in Bangkok, Thailand (the Bangkok Tenofovir Study): a randomised, double-blind, placebo-controlled phase 3 trial. Lancet. 2013;381(9883):2083–90. 10.1016/S0140-6736(13)61127-7 23769234

[7] Grant RM, et al. Preexposure chemoprophylaxis for HIV prevention in men who have sex with men. N Engl J Med. 2010;363(27):2587–99. 10.1056/NEJMoa1011205 PMC3079639 21091279

[8] Thigpen MC, et al. Antiretroviral preexposure prophylaxis for heterosexual HIV transmission in Botswana. N Engl J Med. 2012;367(5):423–34. 10.1056/NEJMoa1110711 22784038

[9] Baral S, et al. Burden of HIV among female sex workers in low-income and middle-income countries: a systematic review and meta-analysis. Lancet Infect Dis. 2012;12(7):538–49. 10.1016/S1473-3099(12)70066-X 22424777

[10] Fonner VA et al. *Effectiveness and safety of oral HIV preexposure prophylaxis for all populations.* AIDS (London, England), 2016. 30(12): p. 1973. 10.1097/QAD.0000000000001145 PMC4949005 27149090

[11] Organization WH. Consolidated guidelines on the use of antiretroviral drugs for treating and preventing HIV infection: recommendations for a public health approach. World Health Organization; 2016. 27466667

[12] Eakle R, et al. Motivations and barriers to uptake and use of female-initiated, biomedical HIV prevention products in sub-Saharan Africa: an adapted meta-ethnography. BMC Public Health. 2017;17(1):968. 10.1186/s12889-017-4959-3 PMC5738143 29258455

[13] Were D, et al. Using a HIV prevention cascade for identifying missed opportunities in PrEP delivery in Kenya: results from a programmatic surveillance study. J Int AIDS Soc. 2020;23:e25537. 10.1002/jia2.25537 PMC7325512 32602658

[14] Mugwanya KK, et al. Scale up of PrEP integrated in public health HIV care clinics: a protocol for a stepped-wedge cluster-randomized rollout in Kenya. Implement Sci. 2018;13(1):118. 10.1186/s13012-018-0809-7 PMC6123996 30180860

[15] Faini D, et al. Awareness, willingness and use of HIV pre-exposure prophylaxis among female sex workers living in Dar-es-Salaam, Tanzania. AIDS Behav. 2023;27(1):335–43. 10.1007/s10461-022-03769-4 PMC9852168 35838859

[16] Skovdal M. Facilitating engagement with PrEP and other HIV prevention technologies through practice-based combination prevention. J Int AIDS Soc. 2019;22:e25294. 10.1002/jia2.25294 PMC6643071 31328412

[17] Van der Elst EM, et al. High acceptability of HIV pre-exposure prophylaxis but challenges in adherence and use: qualitative insights from a phase I trial of intermittent and daily PrEP in at-risk populations in Kenya. AIDS Behav. 2013;17:2162–72. 10.1007/s10461-012-0317-8 PMC3690654 23080358

[18] Eakle R, et al. Motivations and barriers to uptake and use of female-initiated, biomedical HIV prevention products in sub-Saharan Africa: an adapted meta-ethnography. BMC Public Health. 2017;17(1):1–23. 10.1186/s12889-017-4959-3 PMC5738143 29258455

[19] Mugo NR, et al. PrEP for Africa: what we have learnt and what is needed to move to program implementation. Curr Opin HIV AIDS. 2016;11(1):80.

[20] Organization WH. Guideline on when to start antiretroviral therapy and on pre-exposure prophylaxis for HIV. World Health Organization; 2015. 26598776

[21] ANTIRETROVIRAL TS. Guideline on when to start antiretroviral therapy and on pre-exposure prophylaxis for HIV. World Health Organization; 2015. 26598776

[22] Organization WH. Consolidated guidelines on HIV prevention, diagnosis, treatment and care for key populations–2016 update. World Health Organization; 2016. 27559558

[23] Busza J, et al. Understanding early uptake of PrEP by female sex workers in Zimbabwe. AIDS Care. 2021;33(6):729–35. 10.1080/09540121.2020.1832192 33043688

[24] Celum CL, et al. Rethinking HIV prevention to prepare for oral PrEP implementation for young African women. J Int AIDS Soc. 2015;18:20227. 10.7448/IAS.18.4.20227 PMC4509892 26198350

[25] Bjärkefur K, de Andrade LC, Daniels B. iefieldkit: Commands for primary data collection and cleaning. Stata J. 2020;20(4):892–915.

[26] Begnel ER, et al. High pre-exposure prophylaxis awareness and willingness to pay for pre-exposure prophylaxis among young adults in Western Kenya: results from a population-based survey. International journal of STD & AIDS; 2020. p. 0956462420912141. 10.1177/0956462420912141 PMC7291793 32208816

[27] Franks J, et al. PrEP for key populations: results from the first PrEP demonstration project in the Democratic Republic of the Congo. AIDS Care. 2022;34(3):359–62. 10.1080/09540121.2021.1969332 PMC10627785 34495772

[28] Mack N, et al. Barriers and facilitators to pre-exposure prophylaxis (PrEP) eligibility screening and ongoing HIV testing among target populations in Bondo and Rarieda, Kenya: Results of a consultation with community stakeholders. BMC Health Serv Res. 2014;14(1):231. 10.1186/1472-6963-14-231 PMC4051373 24886646

[29] Voeten HA, et al. Female sex workers and unsafe sex in urban and rural Nyanza, Kenya: regular partners may contribute more to HIV transmission than clients. Tropical Med Int Health. 2007;12(2):174–82. 10.1111/j.1365-3156.2006.01776.x 17300623

[30] Fraser N, Gorgens-Albino M, Nkongolo J. *Rapid analysis of HIV epidemiological and HIV response data about vulnerable populations in the Great Lakes Region of Africa. Version 2.3*. 2008.

[31] Thomsen SC, et al. A prospective study assessing the effects of introducing the female condom in a sex worker population in Mombasa, Kenya. Sex Transm Infect. 2006;82(5):397–402. 10.1136/sti.2006.019992 PMC2563858 16854997

[32] MacPhail CL, et al. Perceptions of sexual risk compensation following posttrial HIV vaccine uptake among young South Africans. Qual Health Res. 2012;22(5):668–78. 10.1177/1049732311431944 PMC3735355 22218269

[33] Amoran O, Ladi-Akinyemi T. Sexual risk history and condom use among people living with HIV/AIDS in Ogun State, Nigeria. J Sex Med. 2012;9(4):997–1004. 10.1111/j.1743-6109.2011.02602.x 22214434

[34] Milford C, et al. Lubrication and vaginal sex: lubricant use and preferences in general population women and women at risk of HIV. Arch Sex Behav. 2020;49:2103–16. 10.1007/s10508-020-01673-3 32222851

[35] Kennedy CE, et al. Lubricants for the promotion of sexual health and well-being: a systematic review. Sex Reproductive Health Matters. 2022;29(3):2044198. 10.1080/26410397.2022.2044198 PMC8942543 35315312

[36] Kennedy CE, et al. Lubricants for the promotion of sexual health and well-being. Sex Reproductive Health Matters. 2021;29(3):99–120. 10.1080/26410397.2022.2044198 PMC8942543 35315312

[37] Lancaster KE, et al. Preferences for Pre-exposure Prophylaxis Service Delivery Among Female Sex Workers in Malawi: A Discrete Choice Experiment. AIDS Behav. 2020;24(5):1294–303. 10.1007/s10461-019-02705-3 PMC7995177 31720955

[38] Mijiti P et al. Awareness of and willingness to use oral pre-exposure prophylaxis for HIV prevention among HIV-serodiscordant heterosexual couples: a cross-sectional survey in Xinjiang, China. PLoS ONE, 2013. 8(7). 10.1371/journal.pone.0067392 PMC3712999 23874417

[39] Torres TS, et al. Factors associated with willingness to use pre-exposure prophylaxis in Brazil, Mexico, and Peru: web-based survey among men who have sex with men. JMIR public health surveillance. 2019;5(2):e13771. 10.2196/13771 PMC6601256 31210143

[40] Poon AN, et al. Acceptability and willingness of HIV pre-exposure prophylaxis amongst female sex workers in China. AIDS Care. 2019;31(12):1555–64. 10.1080/09540121.2019.1612001 31046413

[41] Gorin EM, et al. Mobility and HIV vulnerabilities among female sex workers in Guinea-Bissau: findings from an integrated bio-behavioral survey. BMC Public Health. 2023;23(1):1856. 10.1186/s12889-023-16744-y PMC10518914 37749506

[42] Gutin SA, et al. Mobility is Associated with higher-risk sexual partnerships among both men and women in co-resident couples in Rural Kenya and Uganda: a longitudinal cohort study. AIDS Behav. 2023;27(5):1418–29. 10.1007/s10461-022-03878-0 PMC10129962 36318427

[43] Camlin CS, et al. Metrics of Mobility by Sex are Associated with HIV Incidence in Rural Kenya and Uganda. AIDS and Behavior; 2025. pp. 1–13. 10.1007/s10461-025-04743-6 PMC12229744 40327268

[44] Quaife M, et al. Preferences for ARV-based HIV prevention methods among men and women, adolescent girls and female sex workers in Gauteng Province, South Africa: a protocol for a discrete choice experiment. BMJ open. 2016;6(6):e010682. 10.1136/bmjopen-2015-010682 PMC4932295 27354071

